# Effects of clinical pathways in the joint replacement: a meta-analysis

**DOI:** 10.1186/1741-7015-7-32

**Published:** 2009-07-01

**Authors:** A Barbieri, K Vanhaecht, P Van Herck, W Sermeus, F Faggiano, S Marchisio, M Panella

**Affiliations:** 1 Department of Clinical and Experimental Medicine, University of Eastern Piedmont 'A. Avogadro', Novara, Italy; 2 Center for Health Services and Nursing Research, Catholic University Leuven, Leuven, Belgium; 3 Department of Public Health, University Politecnica delle Marche, Ancona, Italy; 4 Sainte Rita Hospital Trust, Vercelli, Italy

## Abstract

**Background:**

A meta-analysis was performed to evaluate the use of clinical pathways for hip and knee joint replacements when compared with standard medical care. The impact of clinical pathways was evaluated assessing the major outcomes of in-hospital hip and knee joint replacement processes: postoperative complications, number of patients discharged at home, length of in-hospital stay and direct costs.

**Methods:**

Medline, Cinahl, Embase and the Cochrane Central Register of Controlled Trials were searched. The search was performed from 1975 to 2007. Each study was assessed independently by two reviewers. The assessment of methodological quality of the included studies was based on the Jadad methodological approach and on the New Castle Ottawa Scale. Data analysis abided by the guidelines set out by The Cochrane Collaboration regarding statistical methods. Meta-analyses were performed using RevMan software, version 4.2.

**Results:**

Twenty-two studies met the study inclusion criteria and were included in the meta-analysis for a total sample of 6,316 patients. The aggregate overall results showed significantly fewer patients suffering postoperative complications in the clinical pathways group when compared with the standard care group. A shorter length of stay in the clinical pathway group was also observed and lower costs during hospital stay were associated with the use of the clinical pathways. No significant differences were found in the rates of discharge to home.

**Conclusion:**

The results of this meta-analysis show that clinical pathways can significantly improve the quality of care even if it is not possible to conclude that the implementation of clinical pathways is a cost-effective process, because none of the included studies analysed the cost of the development and implementation of the pathways. Based on the results we assume that pathways have impact on the organisation of care if the care process is structured in a standardised way, teams critically analyse the actual organisation of the process and the multidisciplinary team is highly involved in the re-organisation. Further studies should focus on the evaluation of pathways as complex interventions to help to understand which mechanisms within the clinical pathways can really improve the quality of care. With the need for knee and hip joint replacement on the rise, the use of clinical pathways might contribute to better quality of care and cost-effectiveness.

## Background

The use of hip and knee joint replacement (JR) has been steadily increasing during the last few years [1]. It is also expected that the pressure for use of JR will further increase in healthcare systems worldwide because of the ageing population and the related increased prevalence of osteoarthritis [2,3]. Although JR is a cost-effective treatment both from the clinical and patients' perspective, JR represents a significant cost to hospitals due to the continuous advances in prosthetic design and materials. This could be a critical issue in healthcare systems because of the decline in available funds for public healthcare [1,4,5]. As a result, from a public health perspective, adjustments in the care process are necessary for cost containment without compromising the quality of patient care [6].

Several methodologies to reduce the costs and to improve the management of these patients have been advocated. A major organisational strategy is a clinical pathway [7-12]. Clinical pathways, also known as care pathways or critical pathways, are a methodology for the mutual decision making and organisation of care for a well-defined group of patients during a well-defined period [7,10,13-15].

Although clinical pathways have been used since the 1980s, there is increasing debate about what they are and how they affect patients' care and outcomes. As a consequence their use in healthcare systems in high volume and costly care like JR is still jeopardised and evidence is needed to support public health decision makers in understanding the real impact of this methodology [9,10,15-20].

Therefore, this meta-analysis was performed to evaluate the use of JR clinical pathways when compared with standard medical care. Based on a previous review the impact of clinical pathways was evaluated assessing the major outcomes of in-hospital JR processes: postoperative complications, number of patients discharged at home, length of in-hospital stay (LOS) and direct costs [9].

## Methods

### Literature search

Medline, Cinahl, Embase and the Cochrane Central Register of Controlled Trials were searched using the following medical subject headings (MeSH) related to clinical pathways and joint replacement: critical pathways AND arthroplasty, replacement, hip AND arthroplasty, replacement, knee AND joint prosthesis. Secondly, a non-MeSH search was performed, based on the following search string: ('clinical pathway' OR 'critical pathway' OR 'care map' OR 'clinical path' OR 'multidisciplinary approach') AND (arthroplasty OR replacement OR prosthesis OR joint OR knee OR hip). The search was limited to articles published between 1975 and 2007, because the first clinical pathways in healthcare originated in the 1980s [21]. No language restrictions were used. The details are reported in the methods for identification of studies in the search strategy file (Additional file 1). The authors of relevant studies were contacted for further information. One author replied that the data was not available [22]. Three authors provided the original data [6,10,11]. The other authors did not reply. The review protocol was not published prior to the study.

### Study inclusion/exclusion criteria

Randomised controlled trials (RCT), controlled clinical trials (CCT, including pseudo-randomised and controlled before-after designs), interrupted time series, cohort and case-control studies were included in the meta-analysis. Studies were considered randomised when it was specifically stated in the text, although the method of randomisation was not always adequately described. Trials were defined as pseudo-randomised when individuals were assigned to alternative forms of treatment using quasi-randomised methods of allocation such as alternation, date of birth or case record numbers. All the included studies compared the care provided through the clinical pathways with standard medical care. Studies were included when at least one of the following outcome indicators have been evaluated: frequency of postoperative complications (complications were defined as factors affecting recovery that required re-admission or prolonged hospital stay such as wound infections, chest infections, pulmonary oedema, deep vein thrombosis, joint dislocation and manipulation, pressure ulcers and urinary tract infections), frequency of patients discharged at home (expressed as a rate), LOS (defined as the number of days of hospitalisation from admission/surgery to discharge from the acute hospital; the Weighted Mean Difference (WMD) of the LOS was used in the study as a synthetic measure of the LOS differences observed in the two groups) and direct costs (referred to total cost of acute hospitalisation such as operating room, patient care unit, medications and supplies: in order to compare clinical pathways with usual care the costs were measured in United States dollars (US$) divided by 10,000 and expressed as WMD). One of eight of the included studies did not report the costs in US$ [23]. Therefore its costs were converted using the official exchange rate of the year of the study (year 1998). The costs were adjusted according to the United States inflation rate of the period of the studies (years 1995 to 2000) and the costs were actualised to year 2000 (mean inflation rate = 2.8% per year). Articles that were strictly descriptive (review articles, historical and theoretical articles), articles with no control group, articles that did not assess at least one of the four outcomes and non-specific articles (for example, JR in hip fracture, JR in femoral neck fracture) were excluded. For continuous variables, since means are influenced by extremes of values, the studies that did not report the standard deviations were also excluded from this meta-analysis [16].

### Outcome measures

The purpose of this research was to combine the results of the published studies on clinical pathways for JR in order to have a total vision of the effects of their implementation. Because clinical pathways are a complex intervention to keep the structure, the multidisciplinary team process and the follow-up of the outcomes of a specific care process alive, the results of the meta-analysis were based on the four outcome measures that have been described before (postoperative complications, discharge to home, LOS and costs) [15]. According to the literature the chosen outcomes were potentially the more suitable measures to describe the effect of the clinical pathways for JR among the endpoints available in the included studies.

### Data extraction and quality assessment

The author, the publication year and country, the sample size, the characteristics of the population studied (age, sex, race, primary diagnosis, ASA score, Charlson score, pain score, operation type, etc.), study design, type of control and outcome measures were recorded [24,25]. Each study was assessed independently by one Italian and one Belgian reviewer. Two reviewers screened all the titles, abstracts and keywords of publications identified by the searches to assess their eligibility. The reviewers were blinded to the names of the authors, institution where the work had been carried out, and the journal. Two reviewers independently assessed the methodological quality of all the included studies and recorded the findings.

Discrepancies between reviewers' assessments of the publications, conceptual problems on the pathway intervention or methodological and statistical problems were solved through discussion with the overall research team. The overall international research team included orthopaedic surgeons, public health specialists, clinical pathway experts, biostatisticians and experts in research methodologies. The assessment of methodological quality of the included studies was based on the Jadad methodological approach for RCT and CCT and the New Castle Ottawa Scale for the case-control studies, cohort studies and time interrupted series [26,27]. The Jadad approach is a five-point scale that assigns points to each study on the basis of the quality of the randomisation generation (0–2 points), of the blinding process (0–2 points) and of the description of withdrawals and dropouts (0–1 point). In general a total score of 3 or more points is achieved only by high quality studies. The New Castle Ottawa Scale is a nine-point scale that assigns points on the basis of the process of selection of the cohorts or of the case and of the controls (0–4 points), of the comparability of the cohorts or of the case and of the controls (0–2 points), and of the identification of the exposure and of the outcomes of study participants (0–3 points). All the studies that met the inclusion criteria (see above) but did not get any points from the assessment of the methodological quality were excluded.

### Data analysis

Data analysis abided by the guidelines set out by The Cochrane Collaboration regarding statistical methods [28]. For dichotomous variables, the relative treatment effect was expressed as relative risk with 95% confidence levels (95%CI). For the meta-analysis of continuous variables, the WMD with 95%CI was used. The weighting procedure took the within study variance around the mean into account to calculate the studies' contribution to the overall results. Since means are influenced by extreme of values, this analysis could use the means only if the standard deviations were also provided. A *P*-value < 0.05 was used as the significance threshold. Statistical heterogeneity of the data was quantified using the *I*^2 ^statistic [29]. For all the outcomes the 'random effects' method was used, based upon intention to treat data from individual studies. The results for each measure outcome were presented separately for randomised and non-randomised studies. The results were represented with the Forest plot, which showed the strength of the evidence: in the plot the left-hand column listed the names of the studies, the right-hand column showed the measure of effect (expressed as odds ratios with 95%CI). According to the test the overall meta-analyses were considered to not have any significant effects at the given level of confidence when the overall diamonds overlapped the line of no-effect results. Potential publication bias was assessed using funnel plots. A funnel plot is a scatterplot of the treatment effects estimated from individual studies (horizontal axis) against a measure of study size (vertical axis). Treatment effects were expressed as risk ratio (RR) or weighted mean difference. Measure of study size was expressed as the reciprocal of studies' standard error [30]. All meta-analyses were performed using RevMan software, version 4.2 [31].

## Results

### Description of the studies

**Figure 1:**
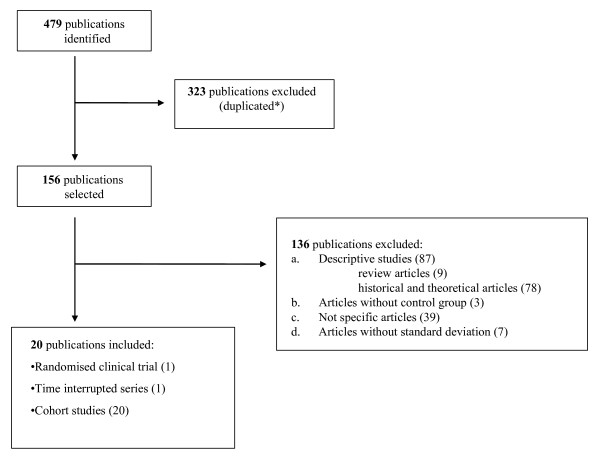
**Flowchart of the selection of the studies**.

The search strategy found a total of 479 records, but only 20 were articles published between 1975 and 2007 and which met the study inclusion criteria; these were included in the meta-analysis for a total sample of 6,316 patients, as shown in Figure 1[6,10,11,22,23,32-46]. The papers of Brunenberg *et al*. [6] and Macario *et al*. [37] reported the results of applying clinical pathways on two cohorts of patients, divided by hip and knee arthroplasty. Because of this we included in the meta-analysis the four studies separately and we coded them as 'Brunenberg Hip', 'Brunenberg Knee', 'Macario Hip' and 'Macario Knee'. So a total of 22 studies, out of the 20 publications selected, were available for the meta-analysis. Two publications reported results of multi-centre design whereas twenty were single-centre studies [see Additional file 2]. In detail the study designs included one RCT [32], one interrupted time series [11] and twenty cohort studies [6,10,22,23,33-46]. Twelve of the twenty-two studies concerned a knee arthroplasty patient group, six a hip arthroplasty patient group and four both. Thirteen studies were based in the United States, two in Australia, one in Belgium, one in Italy, two in The Netherlands, one in New Zealand, one in Spain and one in Taiwan. The setting characteristics of the studies (that is, hospital size and urban/rural typology, education, living situation) were not fully reported.

### Effect of clinical pathways: postoperative complications

**Figure 2:**
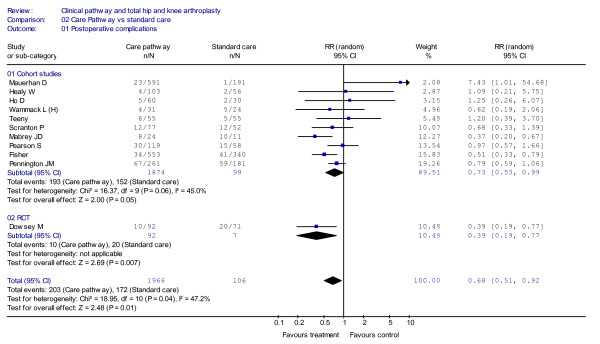
**Meta-analysis of studies evaluating the effect of clinical pathways and standard care on postoperative complications**.

**Figure 3:**
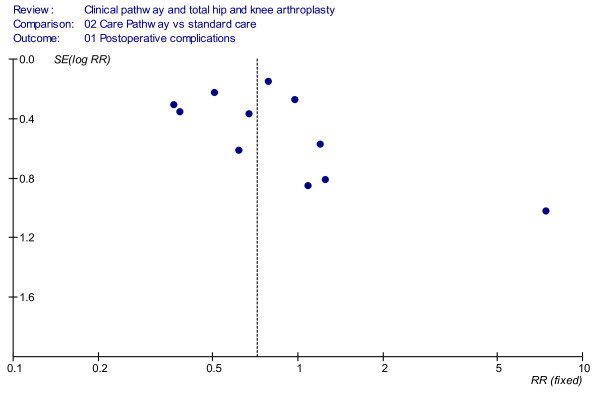
**Funnel plot analyses (postoperative complications)**.

In Figure 2 the results concerning the effect of the implementation of clinical pathways on the incidence of postoperative complications are reported (deep venous thrombosis, pulmonary embolism, manipulation, superficial infection, deep infection, heel decubitus). The aggregate overall results showed significantly fewer patients suffering postoperative complications in the clinical pathways group when compared with the standard care group (RR = 0.68, 95%CI = 0.51–0.92, *P *= 0.01; *I*^2 ^= 47.2 *P *= 0.04). The funnel plot showed a relatively symmetric distribution but not a distinctive funnel form (Figure 3). In detail, in the 10 cohort studies [22,33,34,36,38,40-43,45] that included a total sample of 2,872 patients, a significant trend toward fewer postoperative complications in the clinical pathway group has been shown (RR = 0.73, 95%CI = 0.53–0.99, *P *= 0.05). A significant reduction in complication rates in the clinical pathway group was also shown in the randomised trial by Dowsey *et al*. [32], which included 163 patients (RR = 0.39, 95%CI = 0.19–0.77, *P *= 0.007).

### Effect of clinical pathways: discharge to home

**Figure 4:**
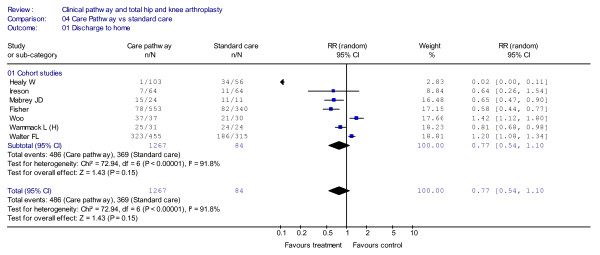
**Meta-analysis of studies evaluating the effect of clinical pathways and standard care on discharge-to-home rates**.

**Figure 5:**
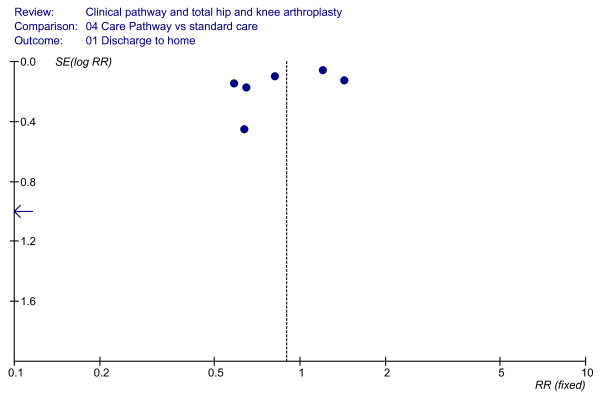
**Funnel plot analyses (discharge to home)**.

As shown in Figure 4, the seven cohort studies [33-36,44-46], which included 2,107 patients, did not report any significant differences in ratios of discharge to home (RR = 0.77, 95%CI = 0.54–1.10, *P *= 0.15; *I*^2 ^= 91.8%, *P *< 0.00001). The funnel plot is shown in Figure 5.

### Effect of clinical pathways: LOS

**Figure 6:**
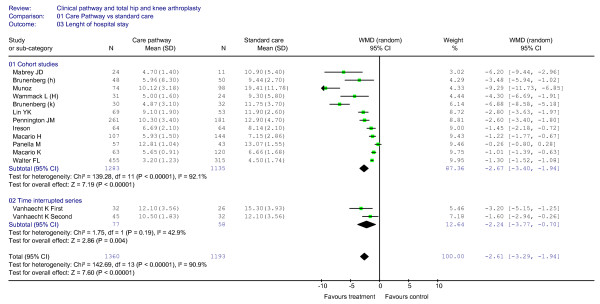
**Meta-analysis of studies evaluating the effect of clinical pathways and standard care on LOS**.

**Figure 7:**
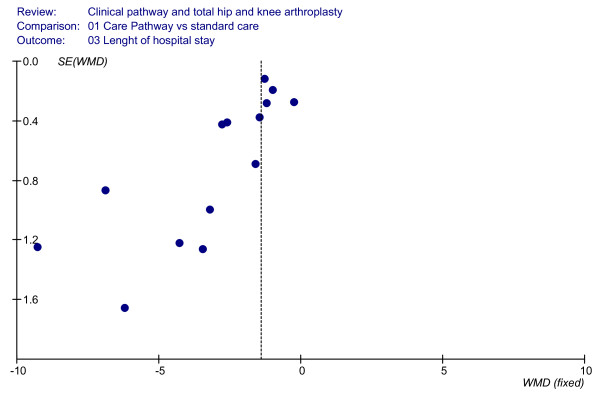
**Funnel plot analyses (LOS)**.

LOS was used as an indicator in 13 studies (aggregated total study sample of 2,553 patients) [6,10,11,23,35-37,39,41,44,45]. As shown in Figure 6, a significantly shorter LOS in the clinical pathway group was observed both in the results of the cohort study designs (WMD = -2.67, 95%CI = (-)3.40–(-)1.94 days, *P *< 0.00001) and of the interrupted time series design [11] (WMD = (-)2.24, 95%CI = (-)3.77–(-)0.70 days; *P *= 0.004). So the overall results showed a significantly shorter LOS for the clinical pathways group when compared with usual care (WMD = -2.61, 95%CI = (-)3.29–(-)1.94 days, *P *< 0.00001; I^2 ^= 90,9%, *P *< 0.00001). The funnel plot is shown in Figure 7.

The studies that did not report the standard deviation [22,32-34,38,40,42,43,46] found a shorter LOS in the clinical pathway group compared with the standard care group (overall mean LOS of 479 days *vs*. 666 days) and in seven studies [22,32-34,38,40,43] the reported differences were strongly significant (*P *< 0.01).

### Effect of clinical pathways: hospitalisation costs

**Figure 8:**
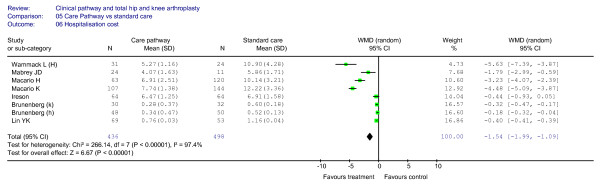
**Meta-analysis of studies evaluating the effect of clinical pathways and standard care on hospitalisation costs**.

**Figure 9:**
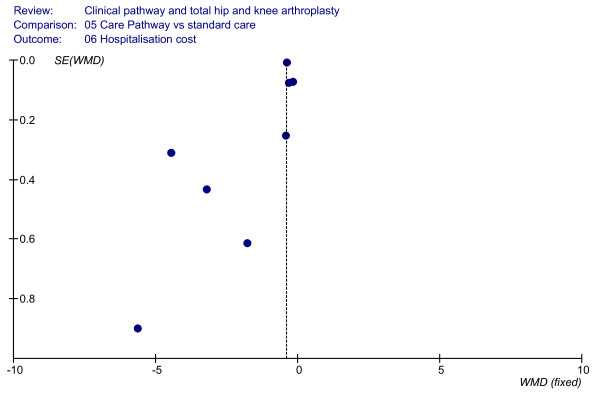
**Funnel plot analyses (hospitalisation costs)**.

The costs during hospitalisation were analysed in eight cohort studies, including an overall sample of 934 patients. These studies [6,23,36,37,45] showed significant differences in hospitalisation costs when comparing the clinical pathways with non-pathway based care. In particular, lower costs during hospital stay were associated with the use of the clinical pathways, as shown in Figure 8 (WMD = (-)1.54, 95%CI = (-)1.99–(-)1.09, *P *< 0.00001; *I*^2 ^= 97,4%, *P *< 0.00001). The relative funnel plot is shown in Figure 9.

Also, in the studies in which the standard deviations were not reported [22,33,34,42] lower mean hospitalisation costs were observed in the clinical pathway group (US$19.401 *vs*. US$22.891) and in three [22,33,34] studies the observed differences were statistically significant.

## Discussion

The main finding that emerged from this meta-analysis is that clinical pathways can effectively improve the quality of the care provided to the patients undergoing JR. The clinical pathways improved the analysed range of selected outcomes (LOS, postoperative complications, discharge to home, hospitalisation cost). We would suggest that this was due to the standardisation of the process of care, even if the knowledge about the mechanisms through which pathways work is insufficient and the evidence determined by meta-analysis is always exploratory in nature and should be considered with caution [47].

A strongly significant reduction in the LOS after implementation of the clinical pathway was observed, and even if it can be argued that a general trend towards a continuous reduction of LOS has been existing in actual systems of care (from 1993 to 1999, the mean hospital stay in acute setting/wards for ankle JR dropped from 6.3 to 4.2 days) [48], this was not observed in the control group. Most of the cohort studies used historical control groups (and therefore potentially susceptible to the bias due to trends in LOS) many authors enrolled consecutive cases in the control groups and this reduced the risk that some cases were missed or excluded, which may have influenced the outcome. Moreover, clinical pathways showed their positive impact on LOS also when applied to other conditions; therefore it is reasonable to think that the reduction of LOS in JR was a consequence of the better organisation of the care when implementing clinical pathways [10,12,18].

The positive effect of clinical pathways on the organisation of the care was also observed in the other measured outcomes. A possible adverse consequence of an overstretched reduction in LOS could have been an increased rate of postoperative complications, because of the reduction of the level of care. The opposite was found in this meta-analysis. The use of clinical pathways significantly decreased the number of postoperative complications, and this was observed for all the complications including deep venous thrombosis, pulmonary embolism, manipulation, superficial and deep infections and heel decubitus, therefore it is possible to conclude that both reduction of LOS and clinical outcome improvements can be attributed to a better organisation of care.

This can also explain the observed reduction in costs while using clinical pathways. An inappropriate process of care can lead to negative clinical outcomes and to a long LOS. This was avoided in the hospitals using clinical pathways. Unfortunately the majority of the studies included in this meta-analysis reported a reduction of hospitalisation costs without specifying the single costs of the specific elements of the process of care, so it is not possible to conclude that the reduction of the costs was achieved by a more appropriate process of care or simply by a generic reduction of the stay. The rate of patients discharged to home was not significantly increased by the use of clinical pathways and this is a possible weakness of the findings.

This meta-analysis has some further limitations. Most of the reviewed studies were performed in academic hospitals and some studies used small sample sizes, therefore a patient selection could have occurred. This could reduce the generalisation of the results but not their strength because patients included in the clinical pathways group did not differ from the patients treated with usual care in age, sex and clinical co-morbidities. Moreover, from a methodological perspective, when evaluating aggregate results, it is easy to forget that most of the included studies were not randomised trials [49]. Despite this, if only one RCT was included in the meta-analysis, the analysed cohort studies showed high quality scores and this helped to ensure the internal validity of the research. The majority of the included studies were performed at single sites, so therefore the same staff could have treated both cases and controls with a possible contamination bias. Adopting part of the pathways in usual care if pathways are effective could simply lead to a reduction of the effects of pathways that in this study remain strongly significant.

As has been reported, the funnel plots showed a relatively symmetric distribution, but the point cloud did not have a distinctive funnel form. This was probably due to the relatively high heterogeneity and to the small number of the primary studies included in the meta-analysis. Therefore a publication bias may have also occurred. This risk is implicit in all meta-analyses or review studies because it is easy to understand that original studies that show no benefit or worse outcome when comparing a new technique with usual care are less likely to be published [47,50]. Two of the included studies [34,42] reported the effects of the clinical pathway together with other hip/knee implant standardisation programmes, and Dowsey *et al*. [32] used pathways in association with a pre-admission information seminar for the patients, which could have further increased the statistical heterogeneity of the results. A random effects analysis was performed in order to control this heterogeneity and to increase the strength of the observed findings [51-53].

The purpose of this study was to give a global vision of the impact of hospital clinical pathways for JR. Some limitations are raised from the nature of clinical pathways that are complex interventions in which is difficult to determine which active components are the determinants of the observed effects [20]. Only a few studies reported on how the clinical pathways were implemented and used at each site, so it is possible that some of the included studies were evaluating different active components with different effects. Moreover, from a health-service research perspective, hospitals are not static environments in which clinical pathways are simply developed and applied but the implementation of clinical pathways is often concurrent with other organisational initiatives that could interact with pathways, enhancing or reducing their effects. It should also be noted that the resources consumed for the development and implementation of clinical pathways were not included in the costs analysis of the studies included in the meta-analysis and this could be a critical issue when applying clinical pathways to low volume hospitals [47].

## Conclusion

Despite the possible limitations, the results of this meta-analysis show that clinical pathways can significantly improve the quality of care. It is not possible to conclude that the implementation of clinical pathways is a cost-effective process, because none of the included studies analysed the cost of the development and implementation of the pathways. The active component of clinical pathways remains unclear in most of the publications. Based on this meta-analysis, the overall pathway literature and the international experience of this research team, we assume that pathways have an impact on the organisation of care if the care process is structured in a standardised way, teams critically analyse the actual organisation of the process and the multidisciplinary team is highly involved in the re-organisation. Further studies should focus on the evaluation of pathways as complex interventions and are needed to further help understand which mechanisms within the clinical pathways can really improve the quality of care.

## List of abbreviations

CCT: controlled clinical trial; JR: joint replacement; LOS: length of in hospital stay; MeSH: medical subject headings; RCT: randomised controlled trial; RR: risk ratio; THA: total hip arthroplasty; TKA: total knee arthroplasty; WMD: weighted mean difference.

## Competing interests

The authors declare that they have no competing interests.

## Authors' contributions

AB and PV searched for and selected the publications. KV and SM extracted and analysed the data. MP conceived of the study and wrote the paper. SW and FF participated in the study design and its coordination and helped to draft the manuscript and discuss the results. All authors read and approved the final manuscript.

## Authors' information

MP is the President of the European Pathway Association, E-P-A , KV is the Secretary General of E-P-A and WS is the treasurer of E-P-A.

## Pre-publication history

The pre-publication history for this paper can be accessed here:



## Supplementary Material

Additional file 1 **Search strategy.** Click here for file

Additional file 2 **Descriptive information of studies included in the meta-analysis.** Click here for file
