# Supplementary material for: Effects of clinical pathways in the joint replacement: a meta-analysis
Source: BMC Med. 2009 Jul 1;7:32. doi: 10.1186/1741-7015-7-32 (PMC2715423; doi:10.1186/1741-7015-7-32)
Supplement: Additional file 1 — Search strategy. [file 1741-7015-7-32-S1.doc]

**Search strategy**

***Types of studies***

All randomized controlled trials (RCTs), quasi-randomized, non randomized controlled clinical trials (CCTs), cohort, case control studies and interrupted time series that compared clinical pathways with standard medical care in patients who undergone joints replacements.

Non-experimental studies, such as the case series design, case studies and case series were not included in the review.

***Types of participants***

We included all studies that recruited patients who had been admitted to hospital for joint arthroplasty. There was no restriction by age, sex, comorbidity.

***Types of intervention***

We sought to assess whether clinical pathways improved outcome compared with standard care. We therefore included any study that had attempted to evaluate such an intervention. We defined a clinical pathway as a methodology for the mutual decision making and organization of care for a well-defined group of patients during a well-defined period.

***Types of outcome measures***

In this review, secondary outcome measures are the other outcome measures that have been reported in the included studies. We sought to include as wide a variety of outcomes as possible in order to describe the full range of potential effects of care pathway care.

These included:

- complications during the hospital stay (e.g. pneumonia, urinary tract infection, deep vein thrombosis, pressure sores);
- number of patients discharged to home;
- duration of hospital stay;
- cost of hospitalisation.

**Search methods for identification of studies**

Relevant trials were identified in the:

- Cochrane Central Register of Controlled Trials (CCRCT) (to June 2007);
- MEDLINE (1975 to June 2007) - since care pathways for healthcare have only existed since the beginning of 1980s, we only searched MEDLINE from 1975 onwards;
- EMBASE (1980 to June 2007);
- CINAHL (1982 to June 2007);

These databases were consulted using OVID search engine. We also handsearched the Journal of Integrated Care Pathways (2001 to 2007), formerly Journal of Managed Care (1997 to 1998) and Journal of Integrated Care (1998 to 2007). We also checked the reference lists of articles retrieved from the above searches and attempted to contact authors of relevant articles where clarification of information was needed.

For the search in MEDLINE, CCRCT were used the following medical subject headings (MeSH) related to clinical pathways and joint replacement: critical pathways AND arthroplasty, replacement, hip AND arthroplasty, replacement, knee AND joint prosthesis. For the search in EMBASE were used the following MeSH: clinical pathways AND arthroplasty, replacement, hip AND arthroplasty, replacement, knee AND joint prosthesis. For the search in CINAHL were used the following CINHAL headings: critical path AND arthroplasty; replacement; hip AND arthroplasty; replacement; knee AND joint prosthesis AND joint prosthesis. Secondly, a non MeSH search was performed, based on the following search string: (“clinical pathway” OR “critical pathway” OR “care map” OR “clinical path” OR “multidisciplinary approach”) AND (arthroplasty OR replacement OR prosthesis OR joint OR knee OR hip).

**Methods of the review**

***Selection of trials***

Two reviewers (BA and VP) screened all the titles, abstracts and keywords of publications identified by the searches to assess their eligibility. The reviewers were blinded to the names of the authors, institution where the work had been carried out, and the journal. Publications that clearly did not meet the inclusion criteria were excluded at this stage. The reviewers excluded articles that did not contain results of any study (e.g. a report simply describing a new care pathway, review articles, historical and theoretical articles), articles with no control group, articles that did not assess at least one of the four outcomes and non specific articles (i.e. JR in hip fracture, JR in femoral neck fracture, JR in fracture kneecap). They resolved any disagreement by discussion.

***Assessment of methodological quality***

Two reviewers (VK and MS) independently assessed the methodological quality of all the included studies and recorded the findings. They noted the important aspects of methodology (e.g. study design, type of control). They used the Jadad score and the New Castle Ottawa Scale to evaluate the methodological quality.

***Data extraction***

One reviewer (VK) extracted the data onto a data extraction form, and the other reviewer (MS) independently checked the extracted data. Data reported in the published sources were used for analyses in this review, but where additional data were needed (e.g. if there were missing data), we attempted to contact the chief investigator of the studies. Disagreement was resolved by discussion and a consensus decision was made.

***Data analysis***

Data analysis abided by the guidelines set out by The Cochrane Collaboration regarding statistical methods. We also consulted a statistician throughout the review. For dichotomous data, we expressed relative treatment effects as relative risk with 95% confidence intervals. For continuous data, we used weighted mean difference with 95%confidence intervals. A p value of less than 0.05 was taken as significant. Heterogeneity between studies was tested using the standard chi squared test. We used a “random effects” method for all outcome measures.
