# Supplementary material for: Effects of clinical pathways in the joint replacement: a meta-analysis
Source: BMC Med. 2009 Jul 1;7:32. doi: 10.1186/1741-7015-7-32 (PMC2715423; doi:10.1186/1741-7015-7-32)
Supplement: Additional file 2 — Descriptive information of studies included in the meta-analysis. [file 1741-7015-7-32-S2.doc]

**Table 1.** Descriptive information of studies included in the meta-analysis.

**Table 1** (continued)
